# The Pivotal Role of TBK1 in Inflammatory Responses Mediated by Macrophages

**DOI:** 10.1155/2012/979105

**Published:** 2012-12-06

**Authors:** Tao Yu, Young-Su Yi, Yanyan Yang, Jueun Oh, Deok Jeong, Jae Youl Cho

**Affiliations:** Department of Genetic Engineering, Sungkyunkwan University, Suwon 440-746, Republic of Korea

## Abstract

Inflammation is a complex biological response of tissues to harmful stimuli such as pathogens, cell damage, or irritants. Inflammation is considered to be a major cause of most chronic diseases, especially in more than 100 types of inflammatory diseases which include Alzheimer's disease, rheumatoid arthritis, asthma, atherosclerosis, Crohn's disease, colitis, dermatitis, hepatitis, and Parkinson's disease. Recently, an increasing number of studies have focused on inflammatory diseases. TBK1 is a serine/threonine-protein kinase which regulates antiviral defense, host-virus interaction, and immunity. It is ubiquitously expressed in mouse stomach, colon, thymus, and liver. Interestingly, high levels of active TBK1 have also been found to be associated with inflammatory diseases, indicating that TBK1 is closely related to inflammatory responses. Even though relatively few studies have addressed the functional roles of TBK1 relating to inflammation, this paper discusses some recent findings that support the critical role of TBK1 in inflammatory diseases and underlie the necessity of trials to develop useful remedies or therapeutics that target TBK1 for the treatment of inflammatory diseases.

## 1. Introduction

Inflammation is the immune response of tissues to pathogens, cell damage, or irritants [1]. It is a protective mechanism used by organisms to remove injurious stimuli. In the process, several symptoms appear, which include redness, swelling, and pain, which are general responses to infection. Inflammation is classified as either acute or chronic. Acute inflammation is the initial response of the organism to harmful stimuli and is induced by the increased movement of plasma and leukocytes from the blood into the injured sites. Chronic inflammation leads to a progressive shift in the type of cells present at the site of inflammation and is characterized by simultaneous destruction and generation of the tissues from the inflammatory process. Inflammation is considered to be the main cause of most chronic diseases including not only inflammatory diseases, such as heart disease, diabetes, Alzheimer's disease, and arthritis, but also cancers [2–5]. Therefore, the study of inflammation should be considered a priority.

The inflammation that occurs during innate immune responses is largely regulated by macrophages [6, 7]. This inflammation is driven by immunopathological events such as the overproduction of various proinflammatory cytokines, including tumor necrosis factor (TNF-*α*), interleukin (IL-1*β*), interferon (IFN-*β*), and several types of inflammatory mediators, including nitric oxide (NO) and prostaglandin E_2_ (PGE_2_) [8]. The production of these inflammatory mediators depends on the activation of pattern recognition receptors (PRRs), including dectin-1, Toll-like receptors (TLR-3 and TLR-4), which are induced by microbial ligands such as lipopolysaccharide (LPS) and polyinosinic:polycytidylic acid (poly(I : C)) [6, 9, 10]. In this signaling process, several kinds of intracellular proteins are activated, which is followed by the activation of transcription factors, such as nuclear factor (NF-*κ*B), activator protein (AP)-1, and interferon regulatory factors (IRF-3 and IRF-7) [6, 9].

A variety of intracellular proteins can initiate the induction of inflammatory responses. TBK1 (TANK {TRAF (TNF (tumor necrosis factor) receptor-associated factor)-associated NF-*κ*B activator}-binding kinase 1), also called NAK (NF-*κ*B activating kinase) and T2K, is a serine/threonine-protein kinase that is encoded by the *tbk1* gene. TBK1 is a member of the I*κ*B kinase (IKK) family and shows ubiquitous expression. It has been demonstrated that TBK1 plays an important role in the regulation of the immune response to bacterial and viral challenges [11, 12]. TBK1 has the ability to regulate the expression of inflammatory mediators such as IL-6, TNF-*α*, and IFN-*β* [11, 13, 14]. Moreover, TBK1 is involved in the insulin signaling pathway, which mediates the phosphorylation of the insulin receptor at serine 994 [15] and is also involved in dietary lipid metabolism [16]. Additionally, activation of the TBK1 signaling pathway could be a novel strategy to enhance the immunogenicity of DNA vaccines [17]. Taken together, these findings suggest that TBK1 acts as a critical player in various immunobiological and immunopathological events, especially inflammatory responses.

Interestingly, TBK1 is expressed in mouse stomach, small intestine, lung, skin, brain, heart, kidney, spleen, thymus, and liver, and at especially high levels in testis [18, 19]. In some inflammatory disease animal models, such as colitis and hepatitis animal models, levels of the active form of TBK1 are elevated compared to nondisease groups (unpublished data). A rheumatoid arthritis animal model has been especially helpful in proving a strong positive relationship between TBK1 and this disease [20]. These observations strongly suggest that TBK1 is closely related to inflammatory diseases. The purpose of this paper is to summarize recent findings and describe the central role of TBK1 in inflammatory response. We hope this paper will provide insight and attract more attention to the study of TBK1 as it relates to inflammation.

## 2. Structure and Function of TBK1

### 2.1. TBK1

**Figure 1:**
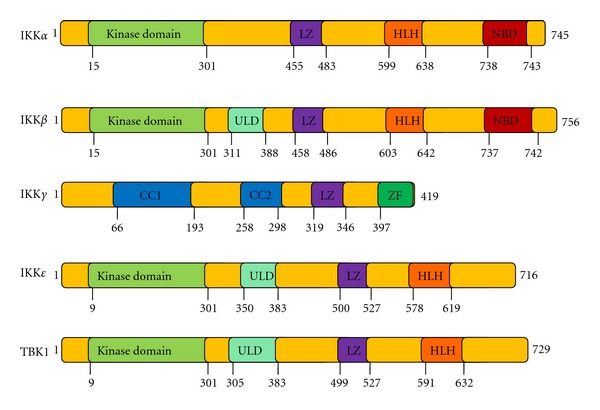
Structural and functional comparisons of the canonical and noncanonical IKKs. KD: kinase domain; HLH: helix-loop-helix; ULD: ubiquitin-like domain; LZ: leucine zipper; CC1, first coiled coil; CC2, second coiled coil; ZF: zinc finger.

TBK1 is a 729 amino acid protein which has four functionally distinct domains; a kinase domain (KD) at the N-terminus, two putative coiled-coil-containing regions in the C-terminal region, including a C-terminal leucine zipper (LZ) and a helix-loop-helix (HLH) motif; a ubiquitin-like domain (ULD) [21, 22] (Figure 1). The ULD is a regulatory component of TBK1 and is involved in the control of kinase activation, substrate presentation, and downstream signaling pathways [21]. The LZ and HLH motifs mediate dimerization, which is necessary for their functions [23].

TBK1 is one of the IKK protein kinase family members that show ubiquitous expression. The IKK family includes two groups: the canonical IKKs such as IKK*α*, IKK*β*, and IKK*γ* (NEMO)  and the noncanonical IKKs such as IKK*ε* and TBK1 (Table 1). Among the members of this family, TBK1 exhibits 49% identity and 65% similarity with IKK*ε*, and IKK*α* and IKK*β* show similar sequence identity [19]. Despite their sequence similarity, TBK1 and IKK*ε* exhibit differential expression patterns. TBK1, like IKK*α* and IKK*β*, is ubiquitously expressed, whereas IKK*ε* expression is restricted to particular tissue compartments, with higher levels detected in lymphoid tissues, peripheral blood lymphocytes, and the pancreas [18, 20]. In addition, LPS and TNF-*α* are also known to activate NF-*κ*B via the involvement of TBK1 and IKK*ε* [24]. Due to these partially overlapping characteristics, TBK1 and IKK*ε* are functionally more similar to each other than to other canonical IKKs [25]. Moreover, mouse and human TBK1 proteins share over 99% homology, indicating that this protein is highly conserved in mammals [18].

### 2.2. TBK1 Substrate Proteins

Studies of the substrate binding capacity of TBK1 have provided great insight into the functions of TBK1. IKK*β* is a direct substrate of TBK1, and is phosphorylated at serines 177 and 181 [18]. Phosphorylation at these sites subsequently induces NF-*κ*B activation and inflammatory responses [18]. Sec5 and DDX3X, which are critical for interferon induction, are also substrates of TBK1 [40, 41]. In addition, AKT (protein kinase B) is a newly-indentified substrate of TBK1. AKT is phosphorylated at serine 476 and activates the IRF3 signaling pathway, which can regulate interferon production and antiviral defense [42, 43]. However, there may be other direct substrates that remain to be discovered, especially in NF-*κ*B-involved signaling pathways since TBK1 strongly induces NF-*κ*B activity [18].

### 2.3. TBK1 Deficiency

TBK1 deficiency could be a direct and effective way to address its functional roles. Macrophages from TBK1-knockout mice show much lower expression levels of IFN-*β* and regulated and normal T cell expressed and secreted (RANTES), as well as decreased IRF3 DNA-binding activity [11]. Mice lacking TBK1 activity exhibit infiltration of immune cells in multiple tissues, including the skin, and increased susceptibility to LPS-induced lethality [11]. TBK1 deficiency also induces massive liver degeneration and apoptosis, along with a dramatic reduction in NF-*κ*B transcription, which indicates that TBK1 is critical in protection against embryonic liver damage due to apoptosis [44]. Furthermore, TBK1 knockout fibroblast-like synoviocytes (FLS) show decreased expression levels of IFN-*β* and IP-10, which are known to contribute to the occurrence of rheumatoid arthritis [20]. This observation indicates that TBK1 could play a significant role in regulating the progression of arthritis [20].

### 2.4. TBK1-Activated Signaling Pathways

**Figure 2:**
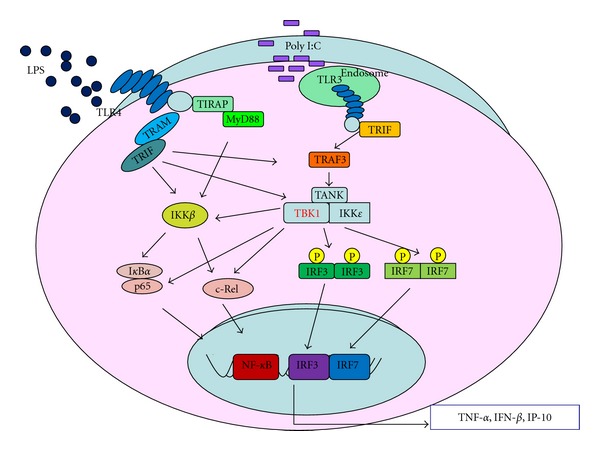
TBK1-regulated signaling pathways in inflammatory responses occurring in activated macrophages.

NF-*κ*B and IRF-3 activated by various TLRs are the major transcription factors involved in the induction of inflammatory mediators, such as NO, PGE_2_, and IFN-*β* [45]. TBK1 is involved in both of these signaling pathways (Figure 2) and is a critical regulator in the interferon response to viral infection [22, 35, 46]. Following activation of TLRs, TBK1 assembles with TRAF3 and TANK to phosphorylate IRF-3, -5, and -7 at multiple serine and threonine residues [47–50]. These IRFs ultimately heterodimerize and translocate into the nucleus, where they induce expression of proinflammatory and antiviral genes such as IFN-*α*/*β* [51, 52]. TBK1 also acts as an NF-*κ*B effector. TBK1 phosphorylates I*κ*B*α* at serine 32, while IKK*ε* phosphorylates serine 36, which induces NF-*κ*B activation [18, 19, 53]. In addition, IKK*β*, RelA/p65, and c-Rel are also phosphorylated by TBK1 at serines 177, 181, and 536, independently, although the phosphorylation site of c-Rel has not yet been confirmed [18, 54, 55]. However, NF-*κ*B activation is for the most part normal in TBK1-knockout mice, and the expression of NF-*κ*B-target genes is only minimally decreased [22]. These results indicate that although TBK1 and IKK*ε* are sufficient, they are not essential for NF-*κ*B activation, and they instead play roles in interferon signaling [22]. 

## 3. TBK1 Functions in Macrophage-Mediated Inflammation

Macrophages are one of the key regulators of the inflammation process. They play critical roles in the initiation, maintenance, and resolution of inflammation [56] and are activated and deactivated during the inflammatory process. Macrophages have three major functions, including antigen presentation, phagocytosis, and immunomodulation through the production of various kinds of cytokines and growth factors [56]. Once macrophages are activated by inflammatory stimuli such as bacteria-derived LPS, pam3CSK, and virus-mimicked poly(I : C), they produce various inflammatory cytokines and mediators, such as those of the IL family, TNF-*α*, interferon *α*/*β*, NO, and PEG_2_ [14, 57–60]. Therefore, macrophages, including RAW264.7 cells and peritoneal macrophages, are generally used in *in vitro* studies of inflammatory responses.

TBK1 is involved in the TLR3 and TLR4 signaling pathways in macrophages [61]. TBK1, especially in the TLR3 signaling pathway, acts as the central kinase directly related to the production of proinflammatory and antiviral cytokines, such as interferon *α*/*β*, IP-10, and RANTES in T cells [62]. IFN-*α*/*β* signaling is critical for host defenses against various types of bacteria, including group B streptococci (GBS), pneumococci, and *Escherichia coli* [63]. IP-10 may play an important role in psoriatic plaques, hypersensitivity reactions, hematopoiesis, and rheumatoid arthritis [18, 64]. RANTES dramatically inhibits the cellular infiltration associated with experimental mesangioproliferative nephritis [65]. In addition, RANTES is the major HIV-suppressive factor produced by CD8 (+) T cells [66]. Furthermore, in a study by Yeh group, TBK1 deficiency was found to result in embryonic lethality; however, this event was rescued by the absence of TNFR, indicating that TBK1 is also involved in TNFR signaling [44]. Moreover, TNF-*α* expression is also dramatically induced in TBK1-overexpressing cells (unpublished data). These findings strongly indicate that TBK1 plays pivotal roles in inflammation, especially in macrophage-mediated systems.

## 4. Development of TBK1-Targeted Drugs as New Immunotherapeutics

### 4.1. The Present Development of TBK1 Inhibitors

The significant role of TBK1 in inflammation has been recently established, and increasing numbers of compounds that target TBK1 for the treatment of inflammatory diseases have been synthesized [67]. BX795 is a well-known TBK1 inhibitor that blocks both TBK1 and IKK*ε* with IC_50_ values of 6 nM and 41 nM, respectively [68]. However, BX795 does not only specifically target TBK1, but also suppresses the activities of other kinases such as PDK1, TAK1, JNK, and p38, even at very low concentrations [68]. A structural understanding of BX795 and information regarding its binding sites, such as its ATP binding motif or other allosteric sites, could allow for the development of new and potent inhibitors specific to TBK1 [6]. For example, MRT67307, which is a new TBK1 inhibitor, derivatized from BX795, suppressed TBK1 activity with much higher specificity (IC_50_ = 19 nM), so that it did not inhibit other kinases such as JNK or p38 [69]. Currently, a number of potential TBK1 inhibitors with high specificity and efficacy are under development by various companies.

### 4.2. TBK1 Inhibitor as a Suppressor of Production of Inflammatory Mediators

Although few specific inhibitors have been developed, large quantities of anti-inflammatory compounds that target TBK1 have been reported (Table 2). For example, resveratrol diminishes the mRNA levels of IFN-1, TNF-*α*, and inducible nitric oxide synthase (iNOS) by inhibiting NF-*κ*B, AP-1, and TBK1/IRF3 signaling pathways [37]. Resveratrol also decreases respiratory syncytial virus- (RSV-) induced IL-6 production in epithelial cells by suppressing TBK1 activity [13]. (−)-Epigallocatechin-3-gallate (EGCG), a flavonoid found in green tea, inhibits COX-2 expression by suppressing MyD88- and TRIF-dependent signaling pathways through direct inhibition of TBK1 activity [39]. Moreover, luteolin and its structural analogues, such as quercetin, chrysin, and eriodictyol, inhibit TBK1 kinase activity and consequently downregulate the expression of TBK1-targeted genes, including TNF-*α*, IL-6, IL-12, IP-10, IFN-*β*, CXCL9, and IL-27 [38].

### 4.3. *In Vivo* Therapeutic Effects of TBK1-Targeted Drugs in Inflammatory Disease Models

Acute and chronic mouse models are generally used in laboratory research for *in vivo* tests. For example, HCl/EtOH can induce gastritis, and dextran sodium sulfate (DSS) and acetic acid can be used to induce colitis [14, 59]. Collagen type has the ability to induce arthritis, and bacteria-derived LPS can induce hepatitis and septic shock. These animal models can be used as representative inflammatory disease models for the testing of drug candidate efficacies [6, 14, 36].

 Currently, only a few studies have been performed to investigate TBK1-targeted treatment of inflammatory diseases. A potential inhibitor of TBK1-mediated signaling pathway is rebamipide [36], which is an amino acid derivative of 2(1H)-quinolinone. Rebamipide is widely used to treat gastric ulcers and gastric injury. In a DSS-induced colitis model, rebamipide treatment showed strong therapeutic effects through the targeting of TBK1-IRF3/7-IFN-*α*/*β* signaling pathways [36]. In addition to this result, our group has also discovered that high levels of active TBK1 are expressed in the DSS-induced colitis model, the HCl/EtOH-induced gastritis model, the LPS-induced hepatitis model, and the collagen type-II-induced arthritis model (unpublished data). Although these results indicate that the TBK1 pathway could be a suitable target for new treatments for inflammatory diseases, only a small number of TBK1 inhibitors have been developed that far. Therefore, increased effort in the development of TBK1-targeted inhibitors is necessary.

## 5. Summary and Perspective

A great number of studies have reported that TBK1 plays pivotal roles in cancers, diabetes, and bacterial and virus infections, especially in inflammatory diseases, including colitis, rheumatoid arthritis, hepatitis, and atherosclerosis. These cumulative studies may provide the essential clues and insights needed for the development of therapeutic strategies against the various diseases involving TBK1. We expect that novel and safe TBK1-targeted drugs or foods with strong efficacy will be developed in the future.

## Figures and Tables

**Table 1:** TBK1, IKK family, and their characteristics.

Subtype	Domains	Phosphorylation site	Sequence identity (%)		Function	Reference
			IKK*α*	IKK*ε*		
Canonical IKKs						
IKK*α*	KD, LZ, HLH, NBD	S176/180	100	27	Osteoclast differentiation; skin tumor suppressor; T-cell receptor signaling pathway; virus response; Toll-like receptor signaling pathway	[26–29]
IKK*β*	KD, ULD, LZ, HLH, NBD	S177/181	52	24	Mediates chemoresistance for cell survival and death; regulates hepatic fibrosis	[30, 31]
IKK*γ*	CC1, CC2, LZ, ZF	S376	None reported	None reported	Regulates I*κ*B kinase (IKK) complex, involved in inflammation, immunity, cell survival	[32, 33]

Noncanonical IKKs						
IKK*ε*	KD, ULD, LZ, HLH	S172	27	100	Regulates type I and type II interferon responses	[34]
TBK1	KD, ULD, LZ, HLH	S172	27	64	Regulates type I and type II interferon responses, mediates NF-*κ*B signaling, liver degeneration, cancer, hepatitis, colitis, and rheumatoid arthritis	[20, 22, 25, 28, 35, 36]

KD: kinase domain; HLH: helix-loop-helix; ULD: ubiquitin-like domain; LZ: leucine zipper; CC1: first coiled coil; CC2: second coiled coil; ZF: zinc finger.

**Table 2:** Naturally occurring compounds targeting TBK1.

Compound	Cells	Action target of TBK1	Reference
Resveratrol	Epithelial cells RAW264.7 cells	Production of IL-6, TNF-*α*, IFN-1, and iNOS	[13, 37]
Luteolin	RAW264.7 macrophage	Expression of TNF-*α*, IL-6, IL-12, IP-10, IFN-*β*, CXCL9, and IL-27	[38]
Quercetin	RAW264.7 macrophage	Expression of IP-10, IFN-*β*	[38]
EGCG	RAW264.7 macrophage	TRIF-dependent signaling pathways	[38, 39]
Chrysin	RAW264.7 macrophage	Expression of IP-10, IFN-*β*	[38]
Eriodictyol	RAW264.7 macrophage	Expression of IP-10, IFN-*β*	[38]

## Abbreviations

TBK1: TANK binding kinase 1
TANK: TRAF (TNF (tumour necrosis factor) receptor-associated factor)
NAK: NF-*κ*B activating kinase
IKK: I*κ*B kinase
PGE_2_: Prostaglandin E_2_
TNF: Tumor necrosis factor
IL: Interleukin
iNOS: Inducible nitric oxide synthase
COX: Cyclooxygenase
TLR: Toll-like receptor
LPS: Lipopolysaccharide
Poly(I : C): Polyinosinic : polycytidylic acid
NF-*κ*B: Nuclear factor-*κ*B
AP-1: Activator protein-1
IKK: I*κ*B*α* kinase
IRF: Interferon regulatory factor
JNK: c-Jun N-terminal kinase
LZ: C-terminal leucine zipper
HLH: Helix-loop-helix
ULD: Ubiquitin-like domain.
